# Insight into the durability of plant resistance to aphids from a demo‐genetic study of *Aphis gossypii* in melon crops

**DOI:** 10.1111/eva.12382

**Published:** 2016-05-13

**Authors:** Sophie Thomas, Flavie Vanlerberghe‐Masutti, Pascale Mistral, Anne Loiseau, Nathalie Boissot

**Affiliations:** ^1^ INRA, CBGPUMR 1062Montferrier‐sur‐LezFrance; ^2^ INRA, GAFLUR 1052AvignonFrance

**Keywords:** agriculture, contemporary evolution, landscape effect, *Vat* gene

## Abstract

Resistance breakdown has been observed following the deployment of plant cultivars resistant to pests. Assessing the durability of a resistance requires long‐term experiments at least at a regional scale. We collected such data for melon resistance conferred by the *Vat* gene cluster to melon aphids. We examined landscape‐level populations of *Aphis gossypii* collected in 2004–2015, from melon‐producing regions with and without the deployment of *Vat* resistance and with different climates. We conducted demo‐genetic analyses of the aphid populations on *Vat* and non‐*Vat* plants during the cropping seasons. The *Vat* resistance decreased the density of aphid populations in all areas and changed the genetic structure and composition of these populations. Two bottlenecks were identified in the dynamics of adapted clones, due to the low levels of production of dispersal morphs and winter extinction. Our results suggest that (i) *Vat* resistance will not be durable in the Lesser Antilles, where no bottleneck affected the dynamics of adapted clones, (ii) *Vat* resistance will be durable in south‐west France, where both bottlenecks affected the dynamics of adapted clones and (iii) *Vat* resistance will be less durable in south‐east France, where only one of the two bottlenecks was observed.

## Introduction

During the 1970s, the concept of ‘durable resistance’ was used to describe resistance that remained effective after deployment at a large scale, over an extended period of time, in ecosystems favourable to the pathogen (Johnson and Law 1975; Nelson 1978). This concept can be used to assess resistance durability only after deployment of the resistance concerned. Thus, starting from the early 1980s, research focused on modelling to obtain an *a priori* estimate of durability of resistance to insects (Gould 1986a,b, 1998) and to pathogens (see van den Bosch and Gilligan (2003) for a review). Apart from *Bt* resistance to lepidopteran populations (Gould 2003; Zhao et al. 2003), the validity of such predictions for natural plant resistance systems has not been documented. As reviewed in Brown (2015), predictions about the durability of resistance are hypotheses about evolution. In this context, the deployment of resistant plants, like the spraying of pesticides, exerts a selective pressure on the demographic features and genetic structure of the targeted pest populations, favouring the selection of individuals adapted to plant resistance. The frequency of adapted genotypes then depends on the frequency of the resistance gene in the agrosystem and on the fitness costs associated with the adaptation. As underline by Brown (2015), estimating cost of virulence is usually challenging especially because cost may vary between different environments. Actually, the effects of plant genotypes on sets of isolates have been frequently characterized under controlled conditions, but such effects have been little investigated at the level of the pathogen or pest population in agrosystems. The most documented example is in the rice/rice blight pathosystem (Vera Cruz et al. 2000), for which field and laboratory studies converged to the role of the fitness cost in adapted blight isolates to explain the durability of the resistance gene *Xa7*.

According to McDonald and Linde (2002), the greatest risk of host resistance being overcome is encountered with pathogens with both sexual and asexual reproduction systems, a high gene flow potential, large effective population sizes and high mutation rates. Aphids, the major arthropod pests of cultivated plants (Dedryver et al. 2010), display most of these features (Hales et al. 1997). Host plant resistance to some aphid species may be efficient and durable, as observed for resistance to phylloxera (*Daktulosphaira vitifoliae*) in *Vitis* (Granett et al. 2001; Korosi et al. 2007). Conversely, resistance‐breaking biotypes have emerged in a number of plant–aphid systems reviewed by Smith and Chuang (2014), but the processes by which plant resistance to aphids is overcome at the population level are poorly understood. Investigations of these processes require knowledge of the genetic control of resistance and the importance of resistance deployment on the one hand and of the biology and structure of the aphid populations targeted on the other. This information is available for the *Aphis gossypii*/melon relationship in France which therefore was chosen as a study case.

The melon‐cotton aphid, *A. gossypii,* is a cosmopolitan, polyphagous species with populations structured into several host races specialized in different crops (Carletto et al. 2009). Each of the host races is distributed worldwide and characterized by a small number of asexual clones, probably because of host plant selection and pest management practices (Carletto et al. 2010; Brévault et al. 2011). One host race is specialized in cucurbits and is by far the most serious pest of Cucurbitaceae crops worldwide. A large genetic survey of winged *A. gossypii* populations visiting melon crops in spring revealed an unexpectedly high level of genetic diversity within the species *A. gossypii* (Thomas et al. 2012a), calling into question the widespread assumption that the melon aphid reproduces exclusively by obligate parthenogenesis in temperate regions (Blackman and Eastop 2007). These winged individuals resulted from a population that occurred at the landscape level, consisting of sexual lineages probably originating from wild plants and asexual lineages specializing on cultivated host plants (Thomas et al. 2012a). The winged aphids giving rise to nymphs on melon crops belonged to the genetic groups containing the asexual lineages specialized in cucurbits. During this sedentary reproduction period, the aphids are wingless but in large colonies, when both the mothers and their offspring experience crowding, winged morphs differentiate allowing dispersal (Dixon 1985).

Aphid resistance in melon (*Cucumis melo* L.) is controlled by the *Vat* gene cluster and quantitative trait loci (QTLs) (Boissot et al. 2010, 2016). In 1987, Margot became the first melon cultivar declared resistant to the melon aphid *A. gossypii* to be listed in the French catalogue. A further 110 Charentais‐type cultivars have since been declared resistant to this aphid in the French or EU catalogues (GEVES data). Using a specific marker of the *Vat‐1* allele, we found that 95% of these cultivars carried resistance to aphids that was probably derived from Margot (GEVES data). In France, melons are cultivated in the south‐east (SE) and south‐west (SW) and on two islands of the Lesser Antilles (LA). Given the commercial success of some of the resistant cultivars, about 80% of the cultivated melon crops in SE France have been assumed to contain this resistance since 2000. Resistant varieties are rarely used in SW France and entirely absent from the LA. In laboratory experiments, the *Vat* cluster present in Margot has been shown to decrease the plant acceptance by *A. gossypii* (i.e. aphids leave the plant after testing) and to decrease the reproductive rate (Boissot et al. 2016). The efficiency of the resistance was found to be dependent on the aphid clone (Thomas et al. 2012a, b; Boissot et al. 2016).

The response of aphid field populations to the selection pressure exerted by *Vat* plants might be expected to depend on their genetic composition, which is influenced by the characteristics of the agrosystem. The present study was carried out over almost 10 years, in three melon‐producing regions with tropical and temperate climates, with and without *Vat*‐mediated resistance deployment and with contrasting cucurbit crops availability. We conducted a demo‐genetic analysis of aphid populations at the crop level taking into account the key phases in the dynamics of crop infestation: visiting by spring migrants (winged), infestation with the wingless nymphs originating from these migrants, development into aphid colonies and the production of winged individuals in large colonies, for dispersal. The aims were (i) to investigate the impact of *Vat* resistance on the density and dispersal of *A. gossypii* populations at the crop level, (ii) to identify resistance‐breaking clones and agrosystem characteristics promoting their emergence and (iii) to infer the durability of *Vat*‐mediated resistance in particular regions and agrosystems.

## Materials and methods

### Field trials

We investigated the *in situ* effect of the *Vat*‐mediated resistance in 21 field trials, using in each trial a pair of melon populations with homogeneous genetic backgrounds with and without resistance (Boissot et al. 2010, 2016) referred to as *Vat* and non‐*Vat* melons, respectively (Table 1). The melon plants were grown from 2006 to 2013, at three sites in SE France (Aramon, Saint‐Andiol and Avignon), one site in SW France (Moissac) and one site in the LA (Petit‐Canal) (Table 1). The study fields were located at INRA experimental units (Avignon and Petit‐Canal) or at the *Centre d'Expérimentation des Fruits et Légumes* (Moissac), or at sites belonging to the seed companies Rijk Zwaan and De Ruiter (Aramon and Saint‐Andiol), who gave permission for the use of their sites and provided technical assistance. All these sites are located in melon‐producing regions under conventional mode of production. Cereals, vineyards and fruit trees occupy most of the SE region, whereas cereals and oleaginous plants are the major crops in the SW. In Guadeloupe (LA), where sugarcane and banana are the major crops, melon crops are located on Grande‐Terre Island, a limestone plateau that regularly experiences severe droughts.

**Table 1:** Characteristics of the field design

Region	Region characteristic Cultivated surface^a^ Climate	Melon cultivation Surface^a^ Period Type Crop cycle length	Location	Coordinates	2006	2008	2009	2011	2012	2013
South‐east France	1.14 10^6^ ha Mediterranean	14 10^3^ ha March – August Greenhouses and open field 130 days	Aramon	43°54′59′’N 04°43′08′’E		1	1			
			Saint‐Andiol	43°50′07′’N 04°56′40′’E		1	2			
			Avignon	43°56′44″N 04°51′52″E	1			1	1	1
South‐west France	4.19 10^6^ ha Oceanic	4 10^3^ ha May to September Open field 150 days	Moissac	44°07′13′’N 01°03′17′’E		1	1	1	1	1
Lesser Antilles	0.65 10^6^ ha Tropical	0.4 10^3^ ha All year long Open field 100 days	Petit‐Canal	16°24′04′’N 61°29′09′’W		1	2	2	1	1

Number of field trials at each site, by year and site characteristics.

a For Lesser Antilles data concerned Guadeloupe and Martinique Islands, both melon producers in the Lesser Antilles.

Melon seeds were sown in a greenhouse, in 60‐mL pots filled with potting soil. The seedlings were transplanted into the field on black plastic mulch after 14–22 days, depending on the trial. They were planted in rows separated by a distance of 2 m, and the plants in each row were 50 cm apart. Drip irrigation was used in all trials. Each trial was divided into two plots of approximately 150 m² each, corresponding to *Vat* and non‐*Vat* melons. Each plot contained 147–208 plants distributed in four to seven rows. *Vat* and non‐*Vat* plots were separated by at least 3–5 m and plants were cut back severely along the border, to prevent the transfer of wingless aphids from plant to plant between the two plots. No insecticides were sprayed during the experiments, except in Petit‐Canal, where the insecticides used only targeted soil or chewing insects. In this region, diazinon (Basudin^®^ Syngenta Agro SAS, Guyancourt, France) was applied a few days before transplantation, to control the mole cricket. *Bacillus thuringiensis* serotype 3 (*Bt*) and diflubenzuron were applied to control *Diaphania hyalinata*, a lepidopteran species that damages leaves. These two insecticides were each applied one to three times during the crop cycle, depending on the year, except in 2011, when *Bt* was applied five times and diflubenzuron was not applied.

### Estimation of *Aphis gossypii* population density and colony occurrence

We estimated sedentary aphid density in the field trials by a qualitative visual counting method (Boll et al. 2002). We tagged 17 areas of 1 m² each (except during the first year, when only eight areas were tagged) per plot, and we then scored aphid density in each area. In total, we monitored aphid density in 288 areas in the SE, 168 areas in the SW and 225 areas in the LA. Four to seven times each crop cycle, we observed 10 leaves in each area and awarded them a score from 0 (no aphids) to 4. Scores of 1 and 2 were assigned to leaves with <10 isolated aphids and small colonies (<50 aphids), respectively. Scores of 3 were assigned to leaves with large colonies (containing up to a few hundred aphids) and scores of 4 were attributed to leaves with larger colonies (some containing more than a thousand aphids) (see Boll et al. (2002) for details relating to this scale). Because large colonies are inducing the production of winged individuals, only areas obtaining a score of at least 3 were considered likely to produce dispersal morphs.

For each area, the mean of the scores assigned to the 10 leaves was calculated and the density index (DI) was calculated as the area under the mean score progression curve. The conditions for normality were respected for ln(DI + 1) in the SE, SW and LA regions, according to the Shapiro–Wilk's test, for an *α* risk of 0.01 (*P *=* *0.09 for the SE and SW data and *P *=* *0.03 for the LA data). We then conducted three independent anovas with the ln(DI + 1) values obtained for the trials in the SE, SW and LA regions, analysing the *Vat* effect nested within the trial effect. If residuals after anova did not fit normality, we calculated the median of DI obtained for *Vat* and non‐*Vat* plants for each trial in the region. A Friedman test was then conducted on paired medians.

### *Aphis gossypii* sampling, DNA analysis and multilocus genotype assignment

We sampled spring migrant populations from 2004 in the SE and 2008 in the SW to 2015, as described by (Thomas et al. 2012a), on melon crops including the field trials described above, except for the SW in 2011. The details of the sampling, corresponding to a total of 2251 aphids, are presented in the supplementary materials (Table S1).

In the field trials, wingless aphids were sampled weekly for 6 weeks, beginning 3 weeks after planting, in each trial. We collected 26–121 wingless individuals per plot, corresponding to either singletons or an individual from a colony produced by a single asexual founding mother (foundress), resulting in a total of 3611 aphids. Each colony was tagged to prevent resampling.

DNA was extracted from each individual aphid with a 5% (w/v) Chelex resin solution, as previously described (Fuller et al. 1999). We amplified eight microsatellite loci specific to the *A. gossypii* genome (Vanlerberghe‐Masutti et al. 1999) in two PCRs, as previously described (Carletto et al. 2009). The amplification products were analysed on an ABI capillary sequencer, with the SeqGen platform (CeMEB LabEx, Montpellier, France). The size of the allele at each locus was determined by comparison with a molecular size standard in GeneMapper v3.7 software (Applied Biosystems, Foster City, California, USA), and a multilocus genotype (MLG) was subsequently assigned to each aphid.

### *Aphis gossypii* diversity analyses

The MLGs identified in the different populations were analysed with the Bayesian software program STRUCTURE (Pritchard et al. 2000). We used an admixture model with a burn‐in of 500 000 and a subsequent Markov chain of 250 000 iterations. For each putative number of clusters (*K*; varying from 1 to 10), 10 replicate runs were compared to assess the consistency of the estimated values. We used the Evanno method to determine the most likely number of genetic clusters (Evanno et al. 2005). For the two most probable numbers of clusters, we used the admixture model for one run, with a burn‐in of 500 000 and a subsequent Markov chain of 1 000 000 iterations. We checked the consistency between the two clustering results, by considering the proximity matrix between the percentages of inferred ancestry of individuals within the different clusters (Pearson's coefficient). MLGs were assigned to a given cluster when the percentage of its inferred ancestry to the given cluster was at least 0.75.

#### Genetic diversity of wingless aphids on *Vat* and non‐*Vat* plants

The clonal diversity of an *A. gossypii* population of *N* individuals was calculated from the Shannon–Wiener index: *H *= –Σ_*i*_ *p*_*i*_ ln *p*_*i*_, where *p*_*i*_ represents the relative frequency of the ith MLG. Clonal diversity is expressed as e^*H*^, as previously described (Vanoverbeke and De Meester 1997), to account for the number of individuals in the sample and the evenness of the relative abundance of the different MLGs. The e^*H*^ values ranged from 1 (i.e. all individuals have the same MLG) to *N* (i.e. all individuals have a different MLG). We used a bootstrap procedure to estimate the standard error of *H*. We created balanced sets, using the *N* bootstrapped data: *N* values were randomly selected with replacement (i.e. each sampled aphid was returned to the data pool before another aphid was sampled). Random sampling and the calculation of *H* were repeated 30 times. For each trail, we calculated the mean e^*H*^ of aphid populations collected on *Vat* and non‐*Vat* plants and a Friedman test was then conducted on these paired e^*H*^ means considering all trials of a given area.

#### Analysis of the selective effect of *Vat* on the development of aphid colonies

If colonies develop at random from wingless individuals regardless of their genotype and *Vat* status, then MLG frequencies should be similar between individuals and colonies. We therefore compared the distribution of MLGs between wingless individuals and colonies. We conducted permutation tests (Manly 1991) in each geographic region, considering the entire population collected from *Vat* and non‐*Vat* plants. In the SE, where the genetic composition of aphid populations was shown to be similar at the various sites (Thomas et al. 2012a), we pooled data for all sites. For each geographic region, *N*_Vat_ is the number of wingless individuals collected from *Vat* plants and *n*_Vat_ is the number of colonies collected from these plants. The mean probability, *P*_MLG‐Vat_, was calculated as follows, for each MLG collected from the colonies. We randomly selected 1000 times, without replacement, *n*_Vat_ from the *N*_Vat_ individuals. Individuals with a MLG present in the random sampling but absent in the *n*_Vat_ were grouped, to calculate *P*_other‐Vat_. For each MLG collected from colonies and the ‘other’ group, the square difference between *P*_MLG‐Vat_ and the MLG frequency observed for the collected *n*_Vat_ individuals was calculated and summed to determine the observed deviation. In total, 1000 sampled deviations were obtained according to this process, for 1000 random samplings without replacement of the same number *n*_Vat_ among *N*_Vat_ individuals. We investigated whether MLG distributions differed significantly between wingless populations and colonies, by calculating the *P*‐value of the observed deviation, estimated as the relative frequency of simulated deviations greater than the observed deviation. The same procedure was followed for samples collected from non‐*Vat* plants.

## Results

### Effects of *Vat*‐mediated resistance on *Aphis gossypii* demography

#### Aphid density on *Vat* and non‐*Vat* melon plants

**Figure 1:**
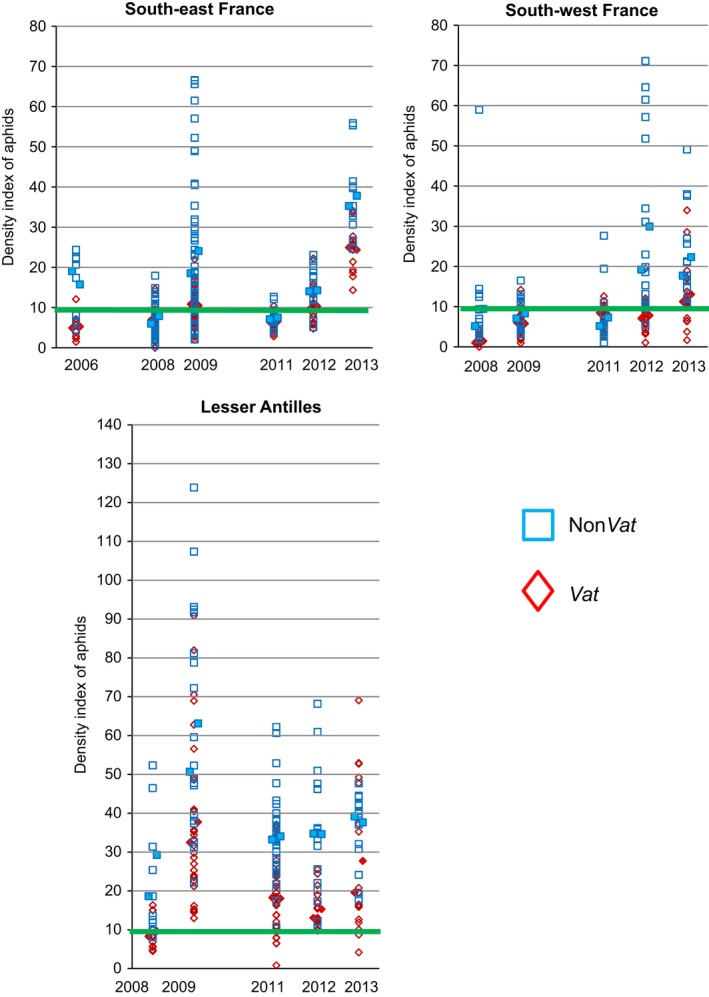
Effect of *Vat*‐mediated resistance on aphid density. The density index (DI) observed in 654 areas (1 m²) in 21 trials, on *Vat* and non‐*Vat* melon plants grown in three melon‐producing regions. For each year, median and mean are shown by full marks on the left and right sides of the data set. The green line indicates the threshold for insecticide application.

On non‐*Vat* plants, *A. gossypii* densities differed between trials, ranging from approximately 3000 aphids/m² in the SW in 2011 to more than 500 000 aphids/m² in the LA in 2013 (Figure S1). Within trials, the highest aphid densities were observed on non‐*Vat* melon plants, except in the SW/2011 trial in which aphid densities were very low on all plants. The *Vat* and trial effects accounted for 64, 57 and 51% of the DI variation (adjusted *r*²) in the SE, SW and LA, respectively. The trials had a significant effect in the SE (*P* = 0.012), but no effect in the SW (*P* = 0.23) and LA (*P* = 0.26). The *Vat* effect was highly significant in each of the three regions (*P *<* *0.0001). Because residuals did not fit normality in the SE, we confirmed the significant *Vat* effect using a nonparametric test (*P* = 0.02). Nevertheless, in the SE, the resistance effect was not strong enough to keep aphid density below the threshold for insecticide sprays in 2009 and 2012, and *Vat*‐mediated resistance was clearly overcome in 2013 (Fig. 1). In the SW, *Vat*‐mediated resistance remained above the threshold value, except in 2013. In the LA, *Vat*‐mediated resistance has been largely overcome since 2009.

#### Estimation of *Aphis gossypii* colony occurrence

**Figure 2:**
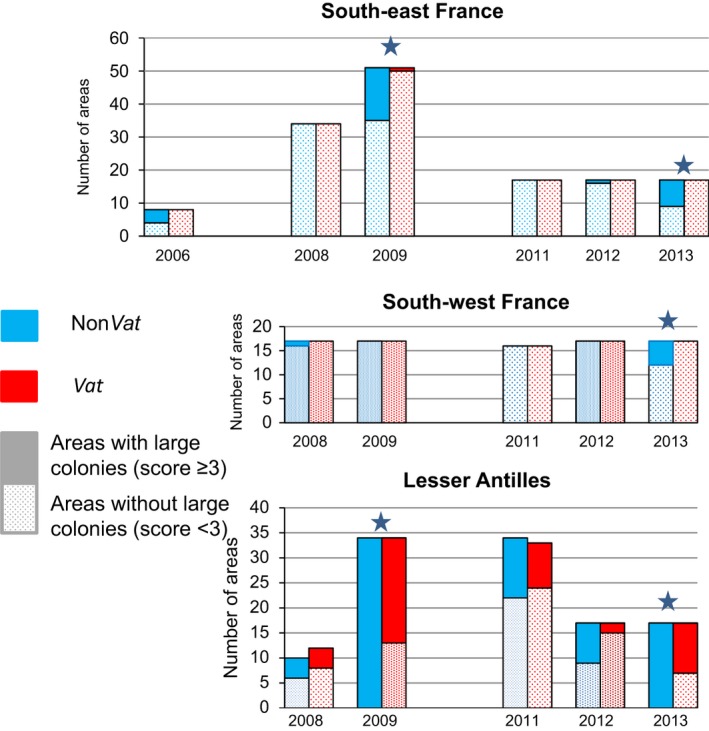
Effect of *Vat*‐mediated resistance on the production of dispersal morphs. Number of 1 m² areas obtaining maximum scores of ≥3 (producing dispersal morphs, plain bars) or <3 (not producing dispersal morphs, spotted bars) in the 21 trials in three melon‐producing regions. Number of areas on *Vat* plants are in red and on non‐*Vat* in blue. Stars indicate significant difference between pairs (*χ* ^2^ test, *P* < 0.05).

The proportion of areas with and without large colonies are shown for each year and region in Fig. 2. This proportion was used as a proxy for the occurrence of the dispersal morph because (i) the production of winged individuals is induced by crowding within the colony and (ii) we observed no nymphs with wing sleeves among the isolated aphids collected (*N *=* *3491) from leaves with a score of <3, from either *Vat* or non‐*Vat* plants. In the SE region, areas of non‐*Vat* plants with large colonies were observed erratically (Fig. 2); thus, dispersal morphs were probably not produced every year. For *Vat* plants, we observed only one area in 1 year with large colonies, suggesting that <1% of the *Vat* plant area produced winged morphs over the 6‐year study period. In the SW trials, some non‐*Vat* plant areas were considered likely to produce dispersal morphs in 2008 and 2013, but none on the *Vat* plants. In the LA, production of winged individuals occurred every year for both *Vat* and non‐*Vat* melon plants, but this happened significantly less often for *Vat* than for non‐*Vat* plants (*χ*² test, *P *<* *0.0001).

### Effects of *Vat*‐mediated resistance on the genetic diversity of *Aphis gossypii* populations

We identified 616 MLGs in the 2251 winged and 3611 wingless *A. gossypii* collected on melon plants in the three regions. They formed seven genetic clusters (Figure S2). We identified 449 MLGs in the winged sample and 230 in the wingless sample. Most of the individuals collected from colonies (99.5%) and most of the wingless individuals (95%) were assigned to three clusters (Figure S2) that were therefore considered to group MLGs belonging to the race specialized in Cucurbitaceae. We named them clusters I, II and III. Conversely, only 78% of the winged individuals were assigned to the Cucurbitaceae race (i.e. clusters I, II or III).

#### Spring migrant populations visiting melon crops

**Figure 3:**
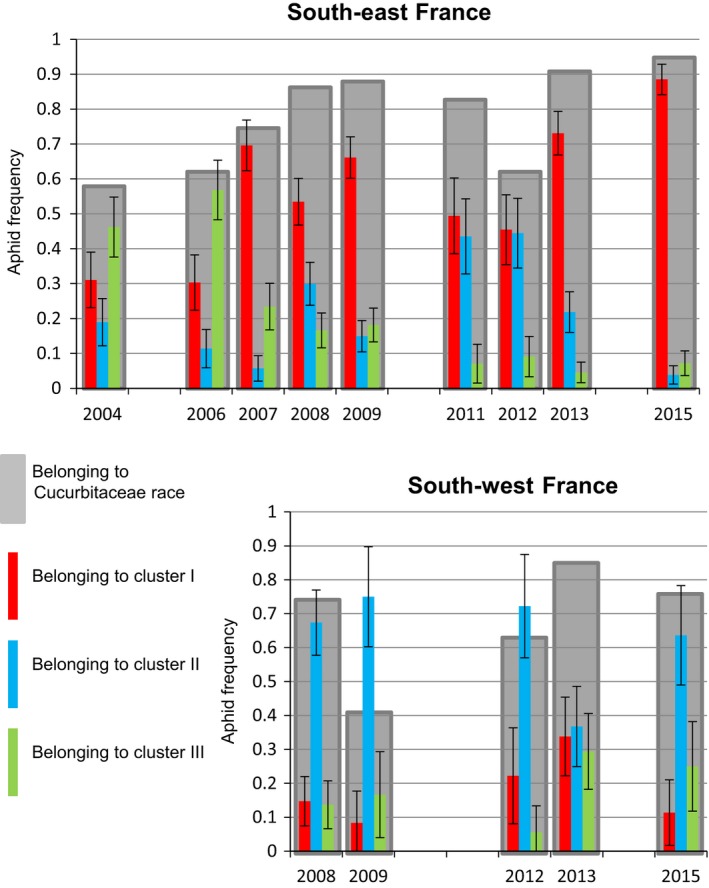
Genetic composition of spring migrant populations over time. Percentage of winged *Aphis gossypii* belonging to the Cucurbitaceae race (grey) collected on melon plants during the 3 weeks after melon transplantation in SE and SW France and their distribution in the genetic clusters I (red), II (blue) and III (green). Bars indicate interval of confidence at *α *= 0.05

In the SE, the percentage of spring migrants belonging to the Cucurbitaceae race varied from 60% to 95% (Fig. 3). Over the years, the frequency of cluster I spring migrants increased, whereas that of cluster III migrants decreased, mostly due to a decline in a particular MLG called NM1. In the SW, the proportion of spring migrants belonging to the Cucurbitaceae race varied from 40% to 85%. Cluster II spring migrants were the most abundant in all years, with no particular trend observed over time.

#### Wingless aphid populations on *Vat* and non‐*Vat* melon plants

Overall, 147 of the 230 MLGs identified in the wingless aphids collected in field trials concerned only one individual. Four MLGs (CUC1, GWD, C6 and NM1) accounted for about 60% of the individuals. Cluster I grouped together 93 MLGs (68% of the individuals), cluster II contained 48 MLGs (14% of the individuals), and cluster III contained 22 MLGs (9% of the individuals). Only 23 MLGs were observed in the LA, and none of them had ever been observed in France. Only 33 MLGs were observed in both the SE and SW regions, and they belonged to the clusters I, II or III.

**Figure 4:**
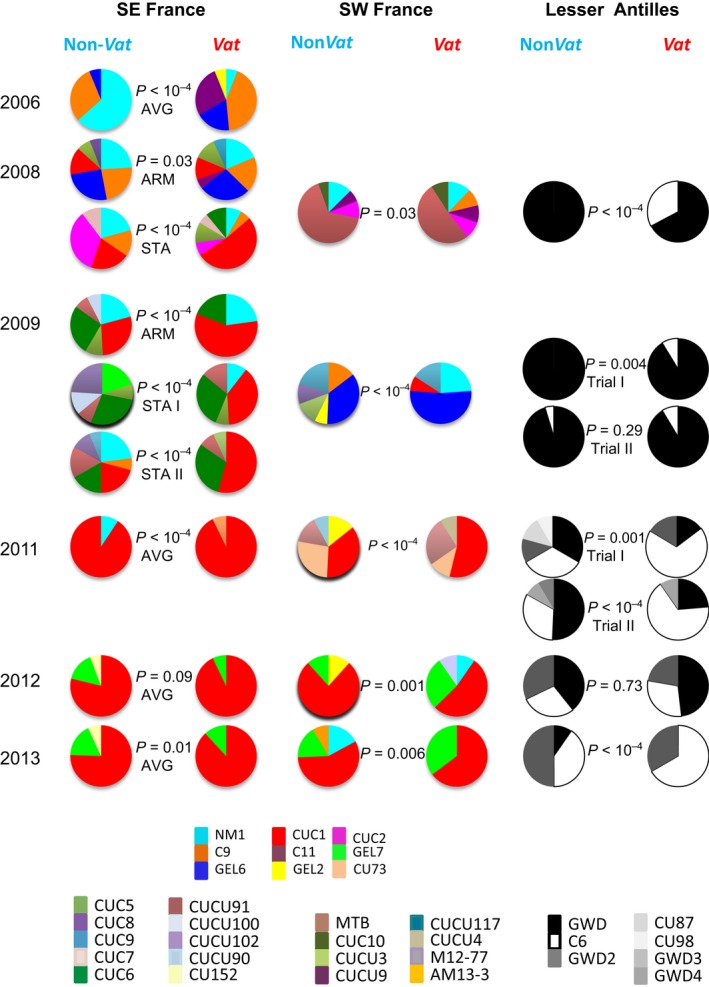
Effect of *Vat‐mediated resistance* on the composition of the wingless aphid population on melon plants. Proportions of individuals collected from non‐*Vat* and *Vat* plants assigned to multilocus genotypes (MLGs) (at frequencies >5%) in 21 trials in the SE (AVG = Avignon, ARM = Aramon, STA = St‐Andiol), SW and the Lesser Antilles. *χ*² tests (Monte Carlo) were used to compare the frequencies of aphid MLGs on *Vat* and non‐*Vat* plants.

In the SE, we identified 114 MLGs in the 1466 individuals collected. No consistent change in the index of clonal diversity was found between paired aphid populations collected on *Vat* and non‐*Vat* plants over the nine trials (Friedman test, *α*_S_ = 0.32, Figure S3). However, when focusing on MLGs with frequencies >5% (Fig. 4), we found significant differences between *Vat* and non‐*Vat* plants in eight of nine trials. The genetic composition of *A. gossypii* populations on both types of melon plant fluctuated from year to year, but some trends were observed. The high frequency of MLGs C9 (cluster I) and NM1 (cluster III) decreased over time, with these MLGs disappearing earlier on *Vat* plants than on non‐*Vat* plants. Conversely, an increase in MLG CUC1 (cluster I), first observed on *Vat* plants in 2008, was observed, with this MLG becoming predominant on both *Vat* and non‐*Vat* plants in 2011. MLG GEL7 (cluster II) emerged in 2011 and was subsequently detected on both *Vat* and non‐*Vat* plants.

In the SW, 125 MLGs were detected in 913 individuals. As in the SE, *Vat* had no consistent effect on the clonal diversity of these populations over the trials (Friedman test, *α*_S_ = 0.66, Figure S3). Nevertheless, the composition of the aphid population was significantly different when *Vat* was present in all trials (Fig. 4). The MLG NM1 was observed every year on non‐*Vat* plants, but only sporadically on *Vat* plants. Consistent with the findings for the SE, two MLGs emerged, CUC1 in 2009 and GEL7 in 2011, with CUC1 subsequently abundant on both *Vat* and non‐*Vat* plants.

In the LA, 23 MLGs were detected in 1232 individuals, and 99.5% of these individuals had a cluster I MLG. Clonal diversity was lower than in the SE and SW (Figure S3). Remarkably, clonal diversity was lower on non‐*Vat* plants than on *Vat* plants until 2009, with the opposite pattern observed during the next 2 years. Only two MLGs, C6 and GWD, were observed until 2009 (Fig. 4). The composition of the aphid population was significantly different on *Vat* plants in only five of seven trials, reflecting a higher frequency of the MLG C6 on *Vat* plants than on non‐*Vat* plants.

We sampled one aphid per colony from 197 colonies in 14 trials: 141 from non‐*Vat* plants and 56 from *Vat* plants. We identified 26 different MLGs in these colonies (Table 2), representing only 10–21% of the MLGs assigned to clusters I, II or III, regardless of the cluster (Monte Carlo test, *P *=* *0.65). The individuals giving rise to colonies had MLGs with a frequency of more than 5% in the wingless population. The Shannon index was lower for colonies than for the wingless populations, for almost all *Vat* and non‐*Vat* plots (Figure S4). We therefore investigated whether colonies developed at random from the wingless population.

**Table 2:** Effect of *Vat*‐mediated resistance on aphid colony development on melon plants

Region	Cluster	MLG	Non‐*Vat* plant		*Vat* plants	
			Collected	Expected	Collected	Expected
South‐east	I	C9	1	1.8		
	I	CUC1	1	7.1	2	1.9
	I	CUC6	12	1.4	2	0.4
	I	CUCU91	2	0.4		
	II	CUCU3	1	0.1		
	III	NM1	4	3.9		
		Others	0	6.3	0	1.7
		Total	21		4	
		*P* ^*a*^	0.01		0.12	
South‐west	NA	C11	1	1.0		
	I	CU103	1	1.0	1	1.0
	I	CUC1	14	11.4	4	5.3
	I	CUC3	1	0.4		
	I	CUCU100			1	0.1
	I	M12‐59	1	0.1		
	II	AM13‐2	1	0.1		
	II	CUCU3	1	1.0		
	II	GEL7	14	4.5	9	2.4
	II	MTB	2	7.2	1	0.8
	II	U13‐39	1	0.1		
	III	M13‐28	2	0.2	1	0.1
	III	NM1	6	5.2	1	1.4
		Others	0	15.5	0	7.8
		Total	48		19	
		*P*	<0.001		<0.001	
Lesser Antilles	I	C6	9	10.2	18	12.3
	I	GWD	35	49.9	15	17.1
	I	GWD2	21	8.0		
	I	GWD3	1	0.8		
	I	GWD4	4	1.2		
	I	M12‐40	1	0.1		
	I	M12‐42	1	0.1		
		Others	0	1.6	0	3.6
		Total	72		33	
		*P*	0.005		0.08	

MLG, multilocus genotype.

Collected: number of individuals, by MLG, sampled from colonies on *Vat* and non‐*Vat* melon plants in three melon‐producing regions. Expected: number of individuals, by MLG, estimated *in silico* from 751 and 715 wingless individuals collected on non‐*Vat* and *Vat* plants in the SE, 500 and 412 wingless individuals in the SW, and 591 and 641 individuals in the Lesser Antilles.

*P* ^*a*^ probability for H0 collected = expected.

In the SE, the MLG composition of the colonies collected from non‐*Vat* plants was significantly different from the expected composition (Table 2); in particular, CUC6 was more frequent than expected. CUC6 was also more frequent than expected on *Vat* plants, although the MLG composition of the colonies on these plants did not differ significantly from the expected composition (permutation test, *P *=* *0.12), probably reflecting the low power of the test due to the small number of colonies considered.

In the SW, the MLG composition of the colonies differed significantly from the expected composition, with GEL7 more frequent than expected on both *Vat* and non‐*Vat* plants (Table 2).

In the LA, the MLG composition of the colonies on non‐*Vat* plants was significantly different from the expected composition, with a higher frequency of GWD2 and a lower frequency of GWD than expected. On *Vat* plants, C6 was observed slightly more frequently than expected (*P *=* *0.08) (Table 2).

## Discussion

According to the definition of a durable resistance (Johnson 1984) largely shared, assessing the durability of a resistance requires long‐term experiments at least at a regional scale. We addressed this issue, by studying aphid population dynamics and genetics on almost isogenic melon plants, differing only in terms of the presence or absence of *Vat*‐mediated resistance to the melon aphid *A. gossypii,* in three regions over almost 10 years equivalent to numerous generations of aphids.

### The effects of *Vat*‐mediated resistance depend on the genetic composition of the aphid populations

As mentioned previously, the dynamics of crop infestation by aphids displays four key phases: visiting by winged aphids, infestation with the wingless nymphs they laid, development into colonies and production of winged individuals for dispersal. The analysis of the dynamics of the first two phases on melon crops revealed a significant decrease in clonal diversity between the spring migrant and wingless populations, reflecting selection by the host plant (Thomas et al. 2012a). We show here that this reduction in clonal diversity continues into the third phase, as only a proportion of the wingless individuals gave rise to colonies (Figure S4). This decrease in diversity, which was particularly large if initial clonal diversity was high, probably reflects differences in fitness or competition between clones on melon plants, as previously observed for *A. gossypii* populations infesting greenhouse cucurbits (Fuller et al. 1999). The densities of wingless populations were reduced on *Vat* plants as compared to non‐*Vat* plants whatever the year and the region (Figure S1), but significant differences were observed between regions.

In the SE and SW, the dynamics of the aphid population on *Vat* plants almost never reached phase 3 (Fig. 2), showing that the containment of the populations by *Vat*‐mediated resistance was sufficient to prevent colony development and, thus, the production of dispersal morphs (Fig. 2). Moreover, in these trials, *Vat*‐mediated resistance significantly selected against aphids assigned to the cluster III (Fig. 4). These findings are consistent with those of a previous laboratory study (Lombaert et al. 2009) showing that *Vat*‐mediated resistance affects the biotic potential of 90% of cluster III clones (NM1) and only 40% of clones assigned to cluster I or II on the basis of their MLG. This selective process has been at work for years in the SE, following the widespread deployment of *Vat* varieties over the agricultural landscape over the last 15 years, as NM1 accounted for 30% of the spring migrant population in 2004 but has not been observed since 2013. As NM1 was the main MLG of cluster III, the decrease in frequency of this MLG drove the decline in this cluster (Fig. 3).

In the LA, *Vat*‐mediated resistance reduced the density of field populations but could not contain the development and dispersal of aphid colonies (Figure S1 and Fig. 2). Genetic diversity was very low in this geographic area, restricted to three major MLGs – C6, GWD and GWD2 – all belonging to cluster I (Table 2). On *Vat* plants, only two MLGs developed large colonies, with C6 displaying a selective advantage over GWD (Table 2). These findings are consistent with those of a laboratory study reporting a greater capacity to colonize *Vat* plants for a C6 clone than for GWD and GWD2 clones (Boissot et al. 2016).

Overall, the results of this study indicate that *Vat*‐mediated resistance affected the *A. gossypii* populations differently in the three regions, due to differences in the genetic composition of the populations visiting the crops, these differences themselves being conditioned by the agrosystem.

### The durability of *Vat*‐mediated resistance depends on regular bottlenecks occurring in the agrosystem

We addressed Mundt's question ‘How do landscape factors influence the population biology of plant pathogens and disease spread?’ raised in a recent review on resistance durability (Mundt 2014). Local selective effects, such as those described above for melon crops during the growing season, may jeopardize the effectiveness of the resistance gene. Nevertheless, local effects may be effaced by gene flow and local extinctions (Burdon and Thrall 1999; Kaltz and Shykoff 2002). In *A. gossypii*, a cosmopolitan pest occurring in various climates and agrosystems, gene flow is dependent on the mode of reproduction and dispersion capacity. Local extinction is dependent on dispersion capacity and resource availability. We inferred the durability of the *Vat‐*mediated resistance in the three agrosystems, taking climate, cucurbit resources and *Vat* deployment characteristics into account, together with aphid population data over a number of years.

The SW and SE regions have several features in common: (i) cold winters with short day lengths that might induce sexual reproduction in *A. gossypii* (Thomas et al. 2012a); (ii) MLGs recurring from year to year (Fig. 4), indicating that a proportion of the population overwinters parthenogenetically; and (iii) erratic production of dispersal morphs on melon crops (Fig. 2). However, the SW and SE agrosystems also displayed several significant differences. In the SE, melon crops account for about 70% of the area under cucurbits grown from January until the end of the autumn, either under cover or in open fields. On the contrary, the melon is the only cucurbit grown at a large scale in the SW, exclusively in open fields, from spring to summer. This difference may explain why spring migrants from the Cucurbitaceae race were less frequent in the SW than in the SE (Fig. 3). Second, the rate of production of dispersal morphs on melon crops was lower in the SW than in the SE (Fig. 2). Thus, aphid populations specialized in melon plants probably experience a higher rate of local extinctions at the end of the cropping season and during the winter in the SW. For example, MTB appeared to be competitive during infestation in 2008, but it was not observed in subsequent years, either among the spring migrants or in the wingless populations. Third, *Vat* varieties have been deployed at a large scale in the SE and imposed a selective pressure on aphid populations for several years, favouring the elimination of clones belonging to the cluster III such as NM1 and the emergence of clones belonging to cluster I and able to multiply on *Vat* plants such as CUC1. CUC1 has been observed every year since 2007 (18% of the spring migrants) and has predominated since 2011 (74% of the spring migrants (Fig. 3). CUC1 did not appear to be highly competitive for the production of dispersal morphs on non‐*Vat* or *Vat* plants (Table 2), but its frequency increased steadily at the landscape level, probably reflecting its considerable overwintering capacity. In the SE, the CUC1 clone is probably jeopardizing the efficacy of *Vat*‐mediated resistance. On the contrary, in the SW, the frequency of cluster III MLGs, such as NM1, was not decreasing with time (Fig. 3), reflecting an absence of recurrent selection against individuals belonging to this cluster in this region where *Vat* varieties have not been deployed. Remarkably, CUC1, the predominant MLG in the SE after 2009, became predominant in the wingless population in the SW after 2011 (Fig. 4). This suggests an expansion of CUC1 from the SE to the SW, probably through the transport of infested plantlets rather than through dispersal flights, although the frequency and efficiency of long‐distance flights by parthenogenetic populations are poorly understood. The deployment of *Vat*‐mediated resistance in the SW might favour the development of this clone, but the combination of a sharp bottleneck at the dispersal morph production stage on crops and heavy local extinctions in the winter may decrease the risk of its expansion.

The LA region has insular agrosystems, in which no dispersion of aphids over long distances is expected, and a tropical climate in which no sexual reproduction occurs. Thus, resource availability and local dispersion of the clones are the key factors to be considered. Cucurbit resources are typically available year‐round, because cucurbit crops are grown throughout the year (pumpkin and chayote in Creole gardens, with melons being the second most abundant fruit produced after the banana). High rates of local extinction were therefore not expected, and no incidences of local extinction were observed in this study. Large numbers of dispersal morphs were produced in this region, with no bottleneck occurring at this level. *Vat* varieties were not deployed in LA, at least until 2011, but two MLGs, GWD and C6, appeared to display pre‐existing adaptation to *Vat*‐mediated resistance, as they were immediately able to develop very large colonies on *Vat* plants. This hypothesis was supported by the detection of C6 since 2002 on Guadeloupe (Carletto et al. 2009). Similarly, virulent biotypes of *Schizaphis graminum* were observed before the deployment of certain resistance genes in wheat (Porter et al. 1997). However, this selective advantage by itself did not allow these virulent biotypes to become predominant in the resistant crops. Conversely, in the LA, *Vat*‐mediated resistance is clearly not durable because *A. gossypii* clones capable of overcoming this resistance expanded as soon as *Vat* plants were grown and lasted over years.

### How can we increase the durability of *Vat*‐mediated resistance?

In the last decade, there has been some application of evolutionary principles to manage plant resistance efficiency through time in order to achieve sustainable disease control in agricultural ecosystems (Thrall et al. 2011; Zhan et al. 2015). The spatial scales also appeared crucial to understanding the evolution of plants and pathogens interactions and landscape influence on evolution of resistance efficiency has been investigated (Papaix et al. 2011, 2015; Fabre et al. 2015). Our results suggest that, for a cosmopolitan pest such as *A*. *gossypii*, decisions concerning resistance deployment should take into account the population genetic structure of the pest at the regional scale. This is consistent with the recent extension of the influenza paradigm to the control of plant pathogens. This paradigm suggests that a knowledge of pathogen population genetics and evolution through continual sampling and monitoring should drive the temporal changes in the deployment of effective resistance genes (Michelmore et al. 2013). However, this strategy of deploying different genes is hampered by the scarcity of resistance genes and a lack of allelic diversity (see examples in Sage‐Palloix et al. (2007); Dogimont et al. (2010)).

In addition, the observations reported here suggest that two processes, the erratic production of small numbers of dispersal morphs on crops and local winter extinction, induce genetic drift and condition the dynamics of populations adapted to *Vat* plants. The manipulation of agrosystems to enhance winter extinction appears unrealistic in the cucurbit/aphid system. In contrast, several strategies could be investigated to avoid production of dispersal morphs. First specific breeding programme could be built to combine several resistance genes. Enhanced genetic drift due to QTLs has recently been shown to contribute to the durability of a major gene conferring resistance to a virus (Quenouille et al. 2013). Similar effects may occur in many plant–pathogen systems and, by analogy, in plant–aphid systems. The use of such a strategy would require the identification of melon QTLs decreasing the production of dispersal morphs on *Vat* plants for use in the breeding programme. Second, insecticide applications to keep aphid population densities under the threshold of winged individual differentiation could be a strategy. This strategy is probably implemented by growers without particular guidance. Third, biocontrol strategy could be associated with *Vat* resistance deployment to keep aphid population levels under the fateful threshold for dispersion occurrence. This strategy could be easily implemented for melons grown in greenhouse where biological control is already used. In open field, biocontrol by conservation is under investigation to decrease the risk of development of aphid colonies on *Vat* plants (Schoeny et al. 2014). Because the combination of different agricultural practices and their spatial and temporal management may lead to local bottlenecks of aphid populations resulting in loss of locally adapted variants, it could play a critical role in determining *Vat* resistance durability.

## Data archiving statement

Data available from the Dryad Digital Repository: http://dx.doi.org/10.5061/dryad.gf54q.

## Supporting information


**Table S1.** Number of winged aphids collected on melon plants 1–3 weeks after transplantation, in sites located in SE and SW France.
**Figure S1**. Effect of *Vat‐*mediated resistance on aphid density.
**Figure S2**. Clustering of the MLGs detected in the aphid populations collected on melon plants.
**Figure S3**. Effect of *Vat‐mediated resistance* on aphid diversity.
**Figure S4**. Effect of *Vat‐mediated resistance* on the diversity of aphids giving rise to colonies.
