# Supplementary material for: Insight into the durability of plant resistance to aphids from a demo‐genetic study of Aphis gossypii in melon crops
Source: Evol Appl. 2016 May 13;9(6):756–68. doi: 10.1111/eva.12382 (PMC4908462; doi:10.1111/eva.12382)
Supplement: Supplementary file 1 — Table S1. Number of winged aphids collected on melon plants 1–3 weeks after transplantation, in sites located in SE and SW France. Figure S1. Effect of Vat‐mediated resistance on aphid density. Figure S2. Clustering of the MLGs detected in the aphid populations collected on melon plants. Figure S3. Effect of Vat‐mediated resistance on aphid diversity. Figure S4. Effect of Vat‐mediated resistance on the diversity of aphids giving rise to colonies. [file EVA-9-756-s001.docx]

**Table S1:** **Number of winged aphids collected on melon plants** 1 to 3 weeks after transplantation, in sites located in SE and SW France

|  | Sitey | **2004** | **2006** | **2007** | **2008** | **2009** | **2011** | **2012** | **2013** | **2015** |
| --- | --- | --- | --- | --- | --- | --- | --- | --- | --- | --- |
| South East France | Aramon |  |  | 72 | 108 | 95 |  |  |  |  |
|  | Avignon | 43 | 206 | 135 |  |  | 102 | 156 | 214 | 218 |
|  | Eyragues | 112 |  |  |  |  |  |  |  |  |
|  | Saint-Andiol | 34 |  |  | 141 | 182 |  |  |  |  |
|  | Valence | 29 |  |  |  |  |  |  |  |  |
|  | Total | 218 | 206 | 207 | 249 | 277 | 102 | 156 | 214 | 218 |
| South West France | Moissac |  |  |  | 123 | 86 |  | 57 | 80 | 58 |

**
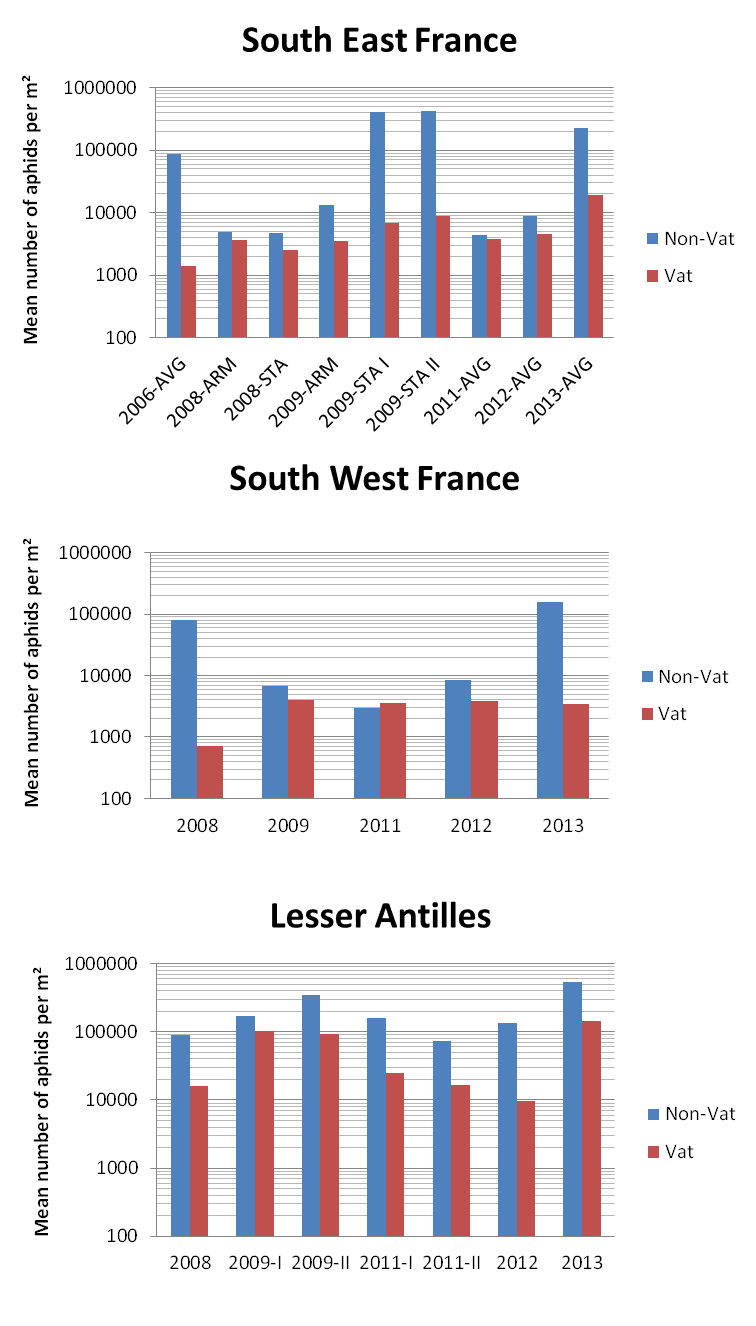
**

**Figure S1: Effect of *Vat-*mediated resistance on aphid density.** Mean number of aphids per m² on *Vat* and non-*Vat* plants in 21 field experiments in three melon-producing regions. AVG for Avignon, ARM for Aramon and STA for Saint-Andiol.

**(a) (b)**


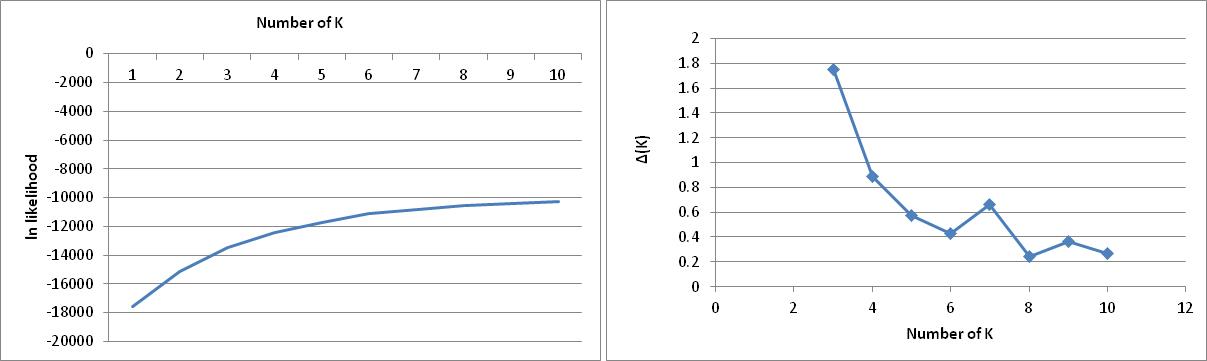
**(c)**

|  | 3(1) | 3(2) | 3(3) |
| --- | --- | --- | --- |
| 7(1) | -0.271 | 0.416 | -0.165 |
| 7(2) | -0.539 | 0.775 | -0.265 |
| 7(3) | -0.285 | -0.234 | 0.653 |
| 7(4) | -0.309 | -0.250 | 0.702 |
| 7(5) | 0.666 | -0.457 | -0.289 |
| 7(6) | 0.158 | -0.040 | -0.152 |
| 7(7) | 0.468 | -0.322 | -0.203 |
| 7(1-2) | -0.657 | 0.961 | -0.343 |
| 7(3-4) | -0.438 | -0.357 | 0.999 |
| 7(5-6-7) | 0.964 | -0.628 | -0.459 |

(d)

|  | 7(1) | 7(2) | 7(3) | 7(4) | 7(5) | 7(6) | 7(7) |
| --- | --- | --- | --- | --- | --- | --- | --- |
| Number of MLGs detected in aphids collected from colonies | 4 | 0 | 0 | 0 | 16 | 0 | 5 |
| **Cluster names given in the manuscript** | III |  |  |  | I |  | II |

**Figure S2: Clustering of the MLGs detected in the aphid populations collected on melon plants.** (a) ln likelihood and (b) delta values for Evanno analysis ([Evanno, Regnaut, and Goudet 2005](#_ENREF_10)) of the results obtained with Structure after the assignment of 616 multilocus genotypes with *K* values from 1 to 10, with 10 simulations of each *K.* (c) Consistency between clustering results for *K* = 3 and *K* = 7, considering the proximity matrix between the percentages of inferred ancestry of individuals within the different clusters (Pearson coefficients). (d) Relationship between the clustering groups and MLGs detected in individuals collected from colonies in melon field trials.

**Figure S3: Effect of *Vat-mediated resistance* on aphid diversity.** Shannon index of diversity for aphid populations collected from *Vat* and non-*Vat* plants during 21 field experiments in three melon-producing regions. AVG for Avignon, ARM for Aramon and STA for Saint-Andiol. Bars indicate the 95% confidence intervals. Effect of *Vat* on e*^H^* was tested by Friedman procedure, SE, α_S_ = 0.32, SO, α_S_ = 0.66, LA, α_S_ = 0.70


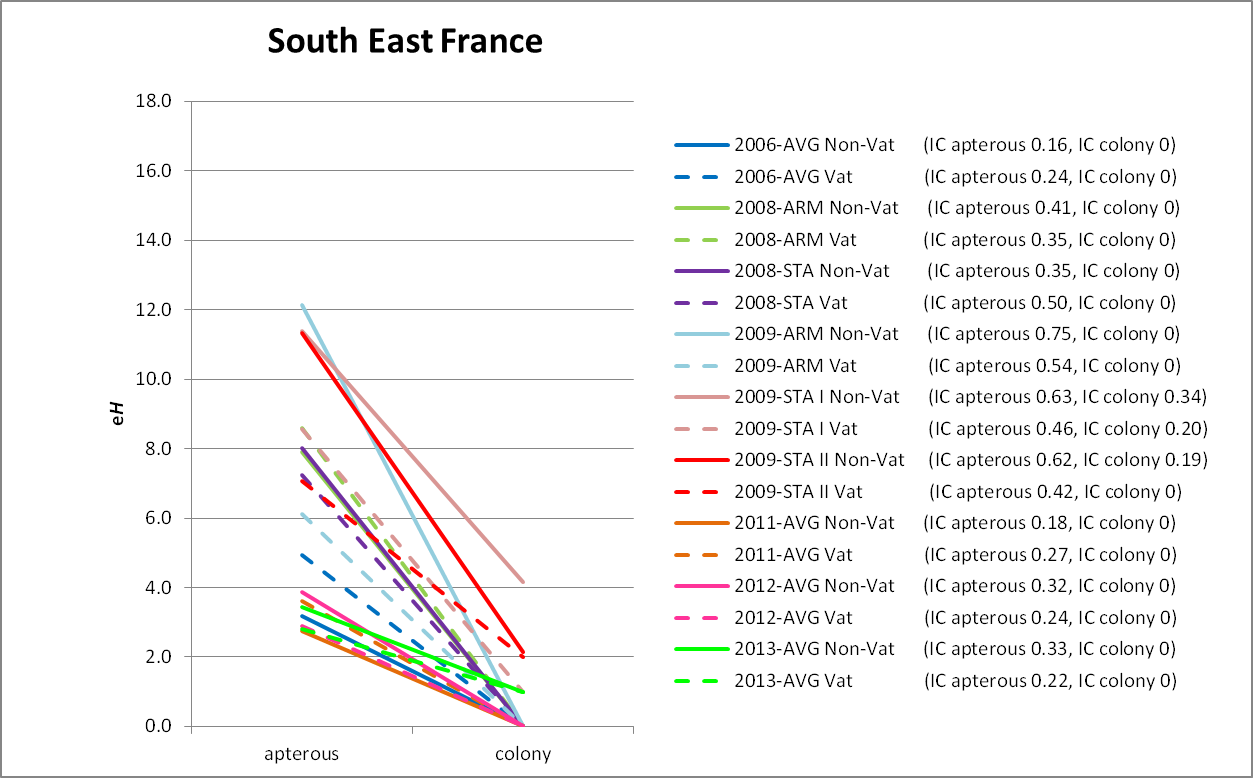
**
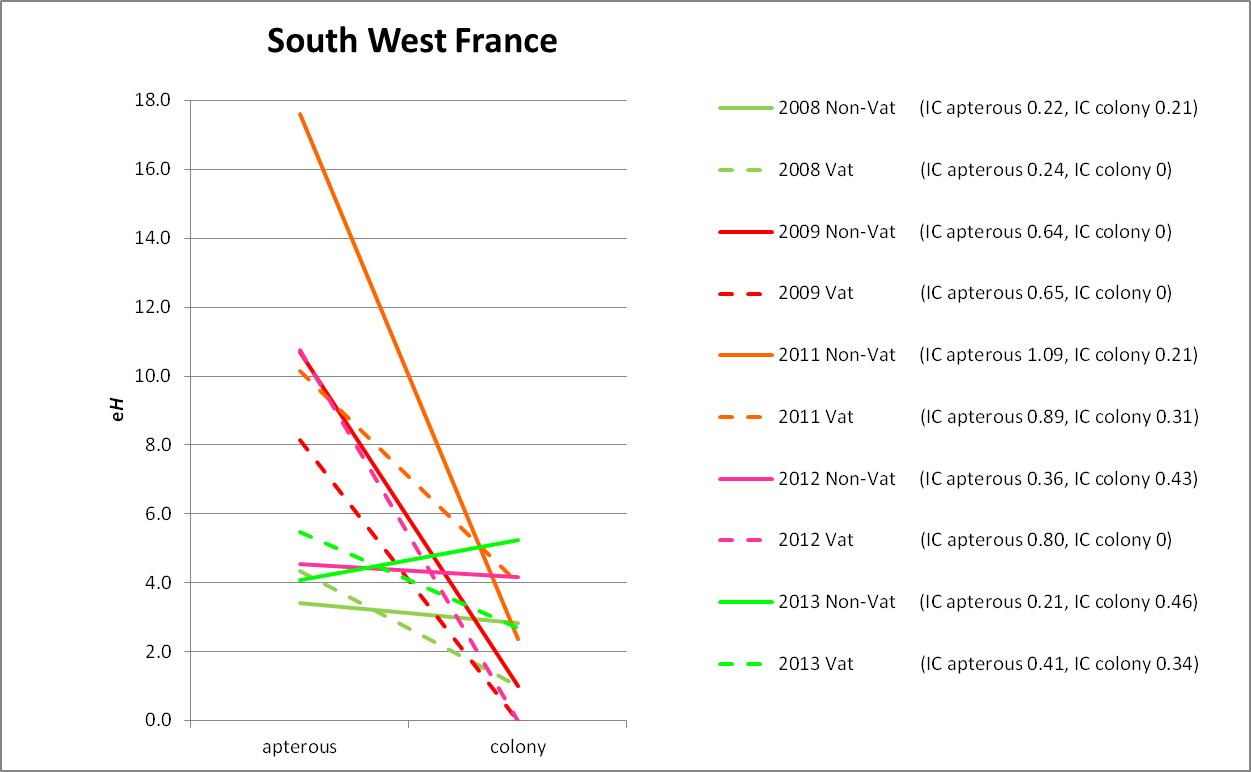

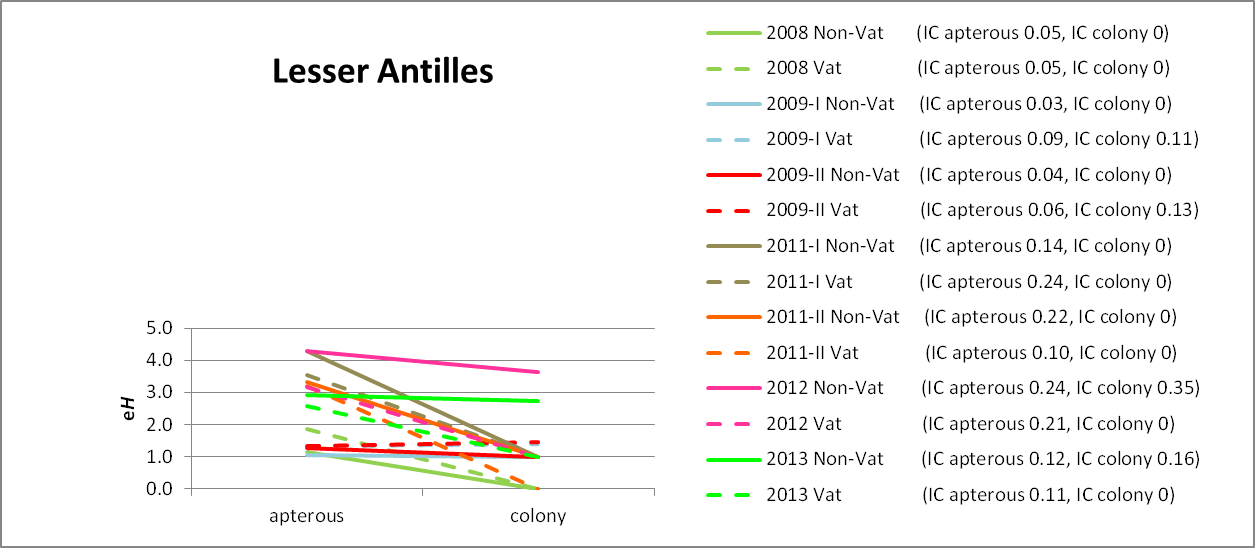
**

**Figure S4: Effect of *Vat-mediated resistance* on the diversity of aphids giving rise to colonies.** Shannon index of diversity for wingless (apterous) populations and colonies collected from *Vat* and non-*Vat* plants in 21 field experiments in three melon-producing regions (IC: interval of confidence at p = 0.05).
